# Protective effect of *Emblica*-*officinalis* in arsenic induced biochemical alteration and inflammation in mice

**DOI:** 10.1186/s40064-015-1227-9

**Published:** 2015-08-21

**Authors:** Manish K. Singh, Suraj Singh Yadav, Rajesh Singh Yadav, Abhishek Chauhan, Devendra Katiyar, Sanjay Khattri

**Affiliations:** Department of Pharmacology, King George Medical University, Lucknow, 226 003 UP India; Department of Criminology and Forensic Science, School of Applied Sciences, Dr. Harisingh Gour Central University, Sagar, 470 003 MP India

**Keywords:** Arsenic, Amla, Inflammation, Immunotoxicity, Mice

## Abstract

Exposure to arsenic in individuals has been found to be associated with immune related problems. In earlier studies, we have demonstrated that amla protects against arsenic induced oxidative stress and apoptosis in thymus and spleen of mice. In continuation to that the present study has therefore been focused to investigate the protective efficacy of amla in arsenic induced inflammation and immunotoxicity in mice. The results showed that arsenic treatment significantly increased serum urea levels (69 %), glucose levels (48 %) and triglyceride levels (66 %) as compared to controls. Mice exposed to arsenic exhibited significant increased in TNF-α (4.3-fold), serum Interleukin-1 beta (threefold), Interleukin-6 (3.8-fold) as compared to controls. Arsenic exposure increased the relative frequency of CD8+ (Tc) cells sub-population (18.9 %) and decreased CD4+ (Th) cells (2.6 %). Arsenic exposure also significantly decreased T (CD3) and B (CD19) cells (21.1 %) as compared to controls. Simultaneously treatment with arsenic and amla significantly inhibited serum urea levels (47 %), glucose levels (50 %) and triglyceride levels (14 %). It also significantly decreased the TNF-α (1.1-fold), levels of IL-1β (1.6-fold), levels of Interleukin-6 (1.3-fold) in serum as compared to those treated with arsenic alone. Simultaneously treatment with arsenic and amla restored the alterations in CD8+ and CD4+ cells and also recovered the damages in B and T sub cells population. Results of the present study clearly indicate that arsenic induced immunotoxicity linked with inflammation has been significantly protected through simultaneous treatment with arsenic and amla that was due to anti-inflammatory and antioxidant activity of amla.

**Electronic supplementary material:**

The online version of this article (doi:10.1186/s40064-015-1227-9) contains supplementary material, which is available to authorized users.

## Background

Arsenic, a naturally occurring metalloid exists in three oxidation states (elemental, trivalent and pentavalent). It is widely present in rocks, soil and ground water due to geochemical composition and its contamination is associated with the global health concern which affects millions of people across the worldwide (Rahman et al. 2010; Yadav et al. 2011; Srivastava et al. 2014). Persons from low economic position and malnourishment have found to be more vulnerable towards the toxic effect of arsenic as such population have mainly depends on the available arsenic contaminated water (Sakurai et al. 2005; Zablotska et al. 2008). After entering the body, arsenic is rapidly undergoes to the metabolic conversion through the process of biotransformation where it converts into toxic arsenite and then transformed into mon-, di-, and trimethylated products (Thomas et al. 2004; Sakurai et al. 2005). Further, it redistributes itself to the entire organ systems of the body and also accumulates in some organs (Ratnaike 2003). Exposure of arsenic to individuals increased the generation of reactive oxygen species and nitric oxide and involved in the production of pro-inflammatory cytokines (Marnett et al. 2003; Islam et al. 2007; Palmieri et al. 2007). Elevated levels of interleukin 6 (IL-6) and tumor necrosis factor alpha (TNFα), produced by the immune system play an important role in increasing the risk of cardiovascular disease. Both IL-6 and TNFα involves in the regulation of the synthesis of other acute phase proteins which could established risk factors for atherosclerosis (Baumann and Gaildie 1994). Alterations in both the intracellular and intercellular homeostasis of these factors leads to possible cell death, inhibition of enzymes, mitochondrial functions and thus enhance the induction of several stress genes (Liu et al. 2001; Singh et al. 2013; Srivastava et al. 2014).

Arsenic exposure in individuals have been reported to cause fetal death due to increased placental inflammatory response, reduced placental T cells and altered levels of cord blood cytokines (Escobar et al. 2010; Ahmed et al. 2011). Arsenic exposure in pregnant may increased the risk of infectious diseases in both mothers and children, indicating arsenic related immunosuppression (Moore et al. 2009; Raqib et al. 2009; Rahman et al. 2010). Immune related disorders associated with poor development of thymus in infants during the pregnancy in arsenic exposed population have also been reported in epidemiological studies (Andrew et al. 2008; Moore et al. 2009; Banerjee et al. 2011). Studies have been reported that chronic exposure to arsenic impairs lymphocyte, monocyte, and macrophage activity and also affects cellular immune responses in mammals, resulting in immunosuppression (Lemarie et al. 2006; Sakurai et al. 2006; Selgrade 2007; Burchiel et al. 2009; Qian et al. 2010). A reduction in CD4+ cell counts, CD4+ :CD8+ cell ratios, and T-regulatory cell levels has been reported in adults and children following exposure to arsenic (McCollum et al. 2005; Hernandez-Castro et al. 2009). Susceptibility of activation of inflammatory cytokines involved in immune related disorders have been reported in humans and rodents exposed to arsenic (Fry et al. 2007).

In recent years, a number of plant products, their active constituents and herbal agents have been used to protect against arsenic induced toxicity (Sharma et al. 2009; Singh et al. 2013, 2014a; Srivastava et al. 2014). The fruit extract of *Emblica officinalis* and its active constituents have long been used in Chinese and Indian traditional system of medicine and has shown anti-oxidative, anti-inflammatory, anti-cancer and immunomodulatory properties (SaiRam et al. 2002; Sreeramulu and Raghunath, 2009; Singh et al. 2013, 2014a). Amla is a rich source of Vitamin C, a water soluble anti-oxidant, a wide variety of phenolics like anthocyanins, flavonols, ellagic acid and its derivatives that acts as a scavenger of free radicals and plays an important role to protect against lipid damage, protein oxidation and DNA oxidation (Sreeramulu and Raghunath 2009; Singh et al. 2013, 2014a). We have recently reported that arsenic induced enhanced oxidative stress linked with apoptosis in thymocytes of mice has been found to be protected through treatment with amla (Singh et al. 2013, 2014a). Arsenic induced hepatic toxicity associated with its accumulation in the liver and impaired antioxidant status has also been found to be protected following simultaneous treatment with arsenic and amla (Singh et al. 2014b). The anti-oxidant potential of amla and its various constituents have been reported but not much is known about its role on inflammatory cytokines in arsenic induced toxicity. Present study has therefore been carried out to understand the protective role of the fruit extract of amla in arsenic induced inflammation and immunotoxicity in mice.

## Methods

### Animals and treatment

The present study was approved by the institutional animal ethics committee of King George Medical University, Lucknow (No. 121 IAH/Pharma-11), India, and all experiments were carried out in accordance with guidelines set by the Committee for the Purpose of Control and Supervision of Experiments on Animals (CPCSEA), Ministry of Environment and Forests (Government of India), New Delhi, India. Male Balb/c mice (15 ± 2 g) were obtained from the animal breeding colony of CSIR-Indian Institute of Toxicology Research, Lucknow. Mice were housed in an air-conditioned room at 25 ± 2 °C with a 12 h light/dark cycle under standard hygiene conditions and had ad libitum access to a pellet diet and filtered water. The dose of fruit extract of *Embilica officinalis* and arsenic is based on our previous findings (Singh et al. 2013, 2014a) for the present study. The mice were randomly divided into four groups with 10 animals/group and the dose of arsenic and amla were given with the help of canola after dissolving in suitable solvent:

- Group I—Mice treated with vehicle (2 % gum acacia) for duration of treatment and served as control.
- Group II—Mice treated with sodium arsenite (dissolved in distilled water at 3 mg arsenic/kg body weight, per os daily for 30 days).
- Group III—Mice treated with fruit extract of *Emblica officinalis* (500 mg/kg body weight, suspended in 2 % gum acacia, *per os* daily for 30 days).
- Group IV—Mice co-treated daily with arsenic and fruit extract as in Groups II and III.

### Blood/tissues collection

At the end of the experimental period (30 days), a set of animals were sacrificed by cervical dislocation. In another set, after heart puncher blood was quickly collected in 10 % EDTA tubes for the separation of serum. For the assessment of different inflammatory markers, the thymus and spleen were isolated from mice following the procedure of Pathak and Khandelwal (2009). The thymus and spleen of five mice/groups were cleaned and placed in phosphate-buffered saline (PBS, pH 7.4) and subsequently processed for measures of immunological parameters. The remaining thymus and spleen in each set were placed in ice-cold saline solution (0.15 M), blot-dried, weighed, and then immediately processed for use in assessment of arsenic level in both tissues.

### Preparation of thymocyte splenocytes suspension

The dissected thymus and spleen from mice and single cell suspension were prepared under aseptic condition. The suspension were passed through stainless steel mesh centrifuged at 200×*g* at 4 °C for 10 min thymocytes resuspended in PBS. Splenocytes were suspended in 5.0 ml hypotonic erythrocyte lysing solution (2.42 g Tris and 7.56 g NH4Cl in 1.0 l deionised water, pH adjusted to 7.2). After 5 min incubation followed by centrifugation, cells were resuspended in PBS, counted through a hemocytometer and then diluted with PBS to appropriate concentrations for use in immunological assays.

### Assay of serum biochemical parameters

Serum samples collected from the blood were subjected to biochemical estimations of serum urea, glucose and TG level and the activity of serum were measured through fully automated biochemical analyzer (CHEMWELL1520, USA). All serum samples were analyzed in duplicate and then mean values were taken.

### Assay of nitric oxide levels

Levels of nitric oxide in serum were estimated using the nitric oxide assay kit procured commercially (Calbiochem, USA) following the method of Misko et al. (1993). The assay principle involves the conversion of nitrate to nitrite by the enzymatic action of nitrate reductase. Addition of 2,3-diaminonaphthalene (DAN) and sodium hydroxide converts nitrite to a fluorescent compound 1(H)-naphthotriazole which is measured spectroflurometrically (excitation 430 nm/emission 450 nm) using multi well plate reader. The results are expressed as p mole nitrate/mg tissue weight.

### Cytokine determination

The parameters related with the cytokine determination have been carried out following the method of Singh et al. (2010a, b).

### Assay of serum Interleukin-1 beta (IL-1β)

The levels of Interleukin-1 beta in serum of mice exposed to arsenic and simultaneous treatment of arsenic and amla were estimated by using the Quantikine mouse IL-1β kit obtained from R&D systems. This assay employs the quantitative sandwich enzyme immunoassay technique (ELISA). An affinity purified polyclonal antibody specific for mouse IL-1β pre-coated plates was used. The intensity of the colour measured was in proportion to the amount of mouse IL-1β bound to the sample.

### Assay of serum Interleukin-6 (IL-6)

The levels of Interleukin-6 in serum of mice exposed to arsenic and simultaneous treatment of arsenic and amla were quantified by using an enzyme linked immunosorbent assay (ELISA) method through the Quantikine mouse IL-6 kit obtained from R&D systems. The intensity of the colour measured was in proportion to the amount of mouse IL-6 bound to the sample. The levels of IL-6 were calculated using a standard curve.

### Assay of serum tumor necrosis factor-alpha (TNF-α)

The quantitative measurements of the level of TNF-α in serum of mice were performed by using the solid phase ELISA. This assay employs the quantitative sandwich enzyme immunoassay technique. The 96-well pre-coated plate with polyclonal antibody specific for mouse TNF-α was used. The intensity of the colour measured was in proportion to the amount of serum TNF-α bound to the sample. The levels of TNF-α were calculated using a standard curve.

### Assessment of T and B lymphocyte population in thymocytes and spleenocytes

T-cells phenotyping were conducted on the basis of CD4 and CD8 surface molecules. Positioning of quadrants on FITC/PE dot plots were performed and CD4+CD8+ (double positive; DP) and CD4−CD8− (double negative; DN) sub-populations were distinguished. The lymphocyte population of splenocytes were assessed based on CD3 (for T-cells) and CD19 (for B-cells) surface molecules. Briefly, cells were suspended in 1 ml PBS. An aliquot of 100 µl was incubated with 5 µl FITC-conjugated anti-CD4 monoclonal antibody and 5 µl PE-conjugated anti- CD8 monoclonal antibody for thymocytes. On the other hand, splenocytes were incubated with 5 µl FITC-conjugated anti-CD3 monoclonal antibody and 5 µl PE conjugated anti-CD19 monoclonal antibody separately for 30 min in dark at room temperature. After incubation, 400 µl PBS was added to each sample. The FITC and PE fluorescence were measured through FL-1 filter (530 nm) and FL-2 filter (585 nm), respectively, on flow cytometer and 10,000 events were acquired. The data were analysed on Cell Quest software and mean fluorescence intensity was obtained by histogram statistics.

### Estimation of arsenic levels in thymus and spleen

Arsenic levels in thymus and spleen of mice exposed to arsenic and simultaneous treatment of arsenic and amla were estimated by hydride system 60 atomic absorption spectrophotometer (HSAAS, ZEEnit 700) following the method of Ballentine and Burford (1957). Briefly, 1 ml of concentrated nitric acid was added followed by 1 ml of perchloric acid to 100 mg of tissue. The sample was then digested over a sand bath until the solution became yellow in colour. If the colour of the digest was brown, more nitric acid and perchloric acid were added and the oxidation was repeated. The digest was made up to known volume with deionized water. Aliquots of this were used to estimate arsenic using atomic absorption spectrophotometer. A calibration curve was constructed by adding known amounts of arsenic standard to calculate arsenic levels in the thymus and spleen regions.

### Statistical analysis

Data were analyzed using one-way analysis of variance (ANOVA) followed by a Newman–Keuls test for multiple pair-wise comparisons among the groups. All values were expressed as mean (±SEM). P < 0.05 was considered significant.

## Results

### Effect on the levels of nitric oxide

**Fig. 1:**
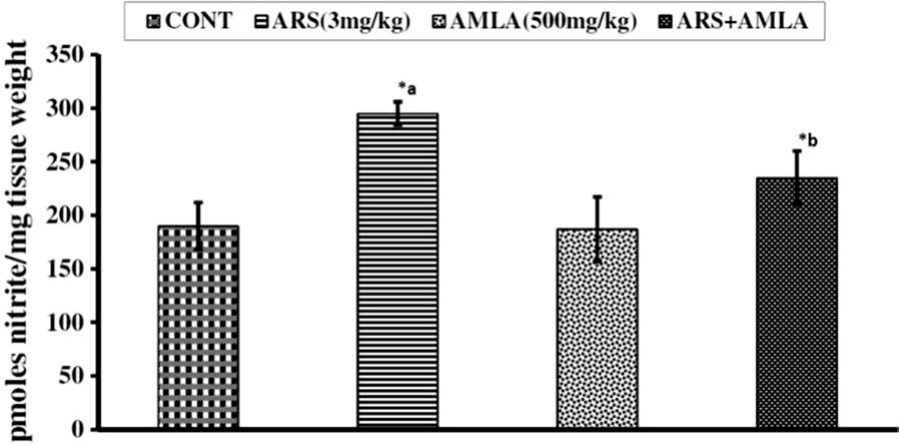
Effect of arsenic, amla and their co-treatment on nitrite levels of serum of mice. Values are mean ± SEM of five animals in each group. *a* Compared to control group, *b* compared to arsenic treated group. *Significantly differs (p < 0.05)

The levels of nitric oxide as a measure of enhanced oxidative stress has been measured and presented in Fig. 1. Mice exposed to arsenic exhibited significant increases in nitric oxide levels (55 %, p < 0.001) as compared to controls. Simultaneous treatment with arsenic and amla significant decreased the nitric oxide levels (20 %, p < 0.05) in serum as compared to those treated with arsenic alone. No significant effect on nitric oxide levels was observed in mice treated with amla alone as compared to controls (Fig. 1).

### Effect on the serum biochemical level in mice

**Fig. 2:**
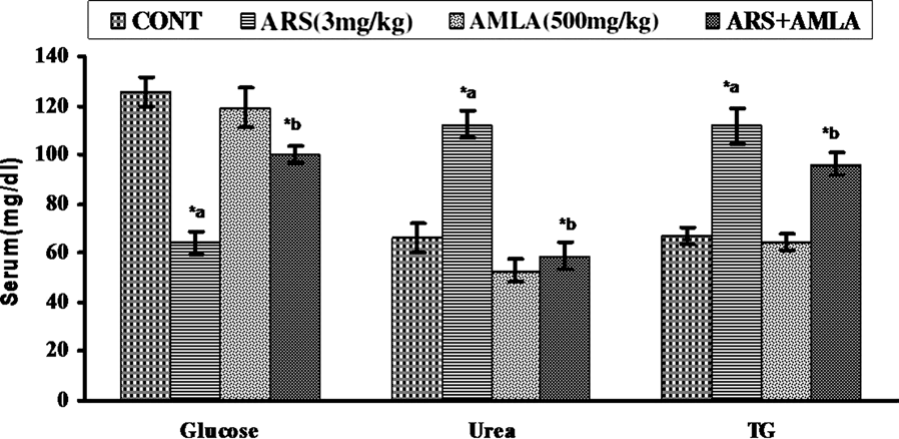
Effect of arsenic, amla and their co-treatment on biochemical estimation in serum of mice. Values are mean ± SEM of five animals in each group. *a* Compared to control group, *b* compared to arsenic treated group. *Significantly differs (p < 0.05)

The effect on the serum biochemical level in mice following exposure to arsenic has been represented in Fig. 2. The results showed that arsenic treatment significantly increased serum urea level (69 %, p < 0.001), glucose level (48 %, p < 0.001), triglyceride (TG) level (66 %, p < 0.001) compared to the control group. Simultaneously treatment with arsenic and amla significantly inhibited serum urea level (47 %, p < 0.001), glucose level (50 %, p < 0.001) and TG level (14 %, p < 0.05) as compared to mice treated with arsenic alone. No significant effect was observed in mice treated with amla alone as compared to controls (Fig. 2).

### Effect on tumor necrosis factor-alpha in serum of mice

**Fig. 3:**
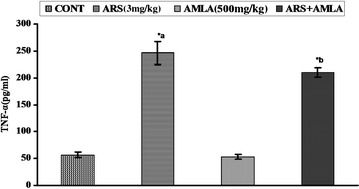
Effect of arsenic, amla and their co-treatment on TNF-α in serum of mice. Values are mean ± SEM of five animals in each group. *a* Compared to control group, *b* compared to arsenic treated group. *Significantly differs (p < 0.05)

Effect of arsenic and co-treatment of arsenic and amla on TNF-α level in the serum has been presented in Fig. 3. Mice exposed to arsenic exhibited significant increases in TNF-α (4.3-fold, p < 0.001) as compared to controls. Co-treatment with arsenic and amla significant decreases the TNF-α (1.1-fold, p < 0.05) in serum as compared to those treated with arsenic alone. No significant effect on serum TNF- α was observed in mice treated with amla alone as compared to controls (Fig. 3).

### Effect on Interleukin-1 beta in serum of mice

**Fig. 4:**
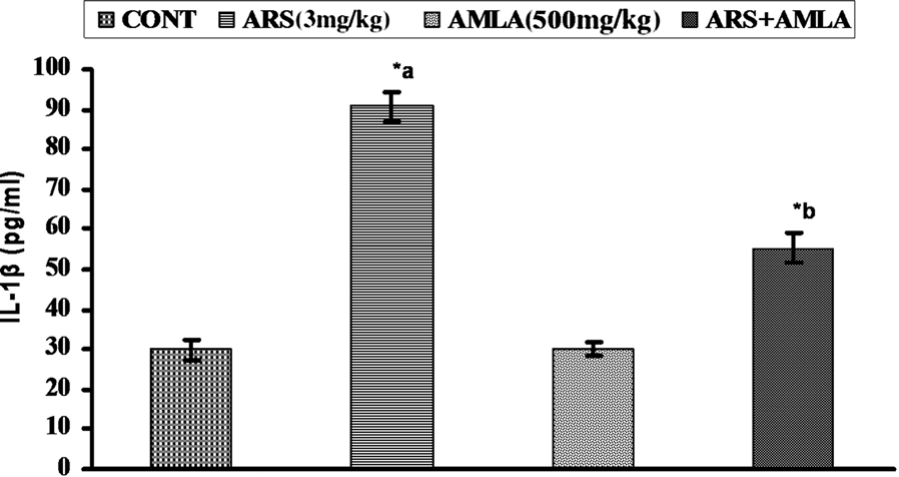
Effect of arsenic, amla and their co-treatment on IL-1β in serum of mice. Values are mean ± SEM of five animals in each group. *a* Compared to control group, *b* compared to arsenic treated group. *Significantly differs (p < 0.05)

Figure 4 indicates the effect of arsenic and co-treatment of arsenic and amla on reduced serum level of interleukin-1β of mice. Exposure to arsenic in mice caused a significantly increases in the levels of serum Interleukin-1 beta (threefold, p < 0.001) in mice as compared to controls. Co-treatment with arsenic and amla decreases the levels of IL-1β (1.6-fold, p < 0.001) in serum of mice as compared to those treated with arsenic alone. No significant effect on the levels of Interleukin-1 beta was observed in the mice treated with amla alone as compared to controls (Fig. 4).

### Effect on Interleukin-6 in serum of mice

**Fig. 5:**
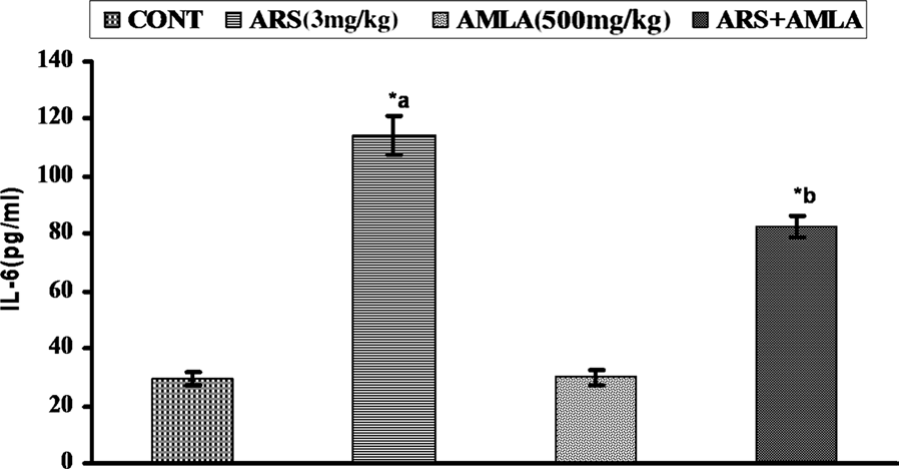
Effect of arsenic, amla and their co-treatment on IL-6 in serum of mice. Values are mean ± SEM of five animals in each group. *a* Compared to control group, *b* compared to arsenic treated group. *Significantly differs (p < 0.05)

Effect of arsenic and co-treatment of arsenic and amla on the serum level of Interleukin-6 in mice has been presented in Fig. 5. Exposure of arsenic to mice caused an increased the serum level of Interleukin-6 (3.8-fold, p < 0.001) as compared to controls. Co-treatment with arsenic and amla decreased the serum level of Interleukin-6 (1.3-fold, p < 0.001) as compared to mice treated with arsenic alone suggested the antioxidant activity of amla. No significant effect was observed in mice treated with amla alone as compared to controls (Fig. 5).

### Effect on T and B lymphocyte population in thymocytes and spleenocytes of mice

The effect of arsenic on thymocyte and spleenocytes population in mice was measured by cell surface marker expression using flow cytometry and expressed in Additional file 1: Table S1. Arsenic exposure increased the relative frequency of CD8 (Tc) cells sub-population from 2.8 to 18.9 % and decreased CD4(Th) cells from 5.3 to 2.6 % as compared to controls. These distinct phenotypic changes were found to be recovered following simultaneous treatment with arsenic and amla. Both CD8 and CD4 cell types came close to controls i.e. 2.5 and 3.4 %, respectively as compared to arsenic treated group. No significant effect on Th cells and Tc cells ratio was observed in mice treated with amla alone as compared to controls group.

Arsenic treated group significantly decreases T (CD3)- and B (CD19)-cells from 35.7 to 21.1 % as compared to controls. Whereas simultaneously treatment with arsenic and amla restored it back to 32.4 %. Similarly, the severely affected B cell population was also restored to 50.3 % from 32.5 %, by fruit extract of amla. No significant effect on the ratio of T/B cells was observed in the mice treated with amla alone as compared to controls group.

### Effect on arsenic levels in thymus and spleen of mice

A significant increase in the levels of arsenic in thymus and spleen (3.5-fold, p < 0.01), was observed in mice exposed to arsenic for 30 days as compared to controls (Additional file 1: Table S2). Levels of arsenic were found to be significantly decreased in thymus and spleen (1.3-fold, p < 0.05), in mice simultaneously treated with arsenic and amla as compared to mice treated with arsenic alone. No significant change in arsenic levels in the thymus and spleen was observed in mice treated with amla alone as comparison to controls (Additional file 1: Table S2).

## Discussion

Clinical and pre-clinical studies showed that arsenic act as an immunosuppressant and its exposure in individuals caused various immunological disorders at molecular and cellular levels (Islam et al. 2007; Biswas et al. 2008). Arsenic is a potent immunotoxicant which modulates non-specific immune responses and alters the expression of cytokines in time and dose dependent manners (Das et al. 2011). Enhanced oxidative stress resulting from the imbalance between pro- and antioxidant systems leads to the disruption of biological membrane and contributes to inflammation and production of pro- inflammatory cytokines (Chang et al. 2007; Singh et al. 2010a, b). Increased generation of free radicals/reactive oxygen species (ROS) associated with enhanced oxidative stress have been found to be implicated in the induction of apoptosis in both patho and physiologic states (Stepnik et al. 2005; Sharma et al. 2009). Arsenic induced depletion of anti-oxidant defence enzymes including superoxide dismutase, catalase, and glutathione peroxidase associated with apoptosis, immunotoxicity and hepatic damage have been recently reported by us (Singh et al. 2013, 2014a, b). In the present study, arsenic treatment in mice significantly increased the levels of nitric oxide in thymocytes and spleenocytes of mice as compared to the controls further indicates the enhanced oxidative stress.

The alterations in the serum urea level considered as a significant marker of renal dysfunctions. The increased breakdown of tissue, dietary or impaired excretion also found to be linked with the high levels of blood urea (Polliack et al. 1993). The increased urea concentration in serum following treatment with arsenic in mice has been considered due to its effect on liver functions (Sands 1999). In human population, chronic arsenic exposure has been showed to increase blood glucose level (Tseng 2004). Enhanced oxidative stress due to arsenic exposure damages the β cells of the pancreatic islets leading to increase blood glucose levels (Hayden and Tyagi 2002). In the present study, arsenic treatment in mice significantly increased the serum urea, glucose and triglyceride level associated with enhanced levels of nitric oxide clearly indicate the increased oxidative burden as compared to the controls are consistent with the earlier studies.

In an epidemiological study, Biswas et al. (2008) reported that chronic arsenic exposure through contaminated drinking water caused immunosuppression and affect the secretion of variety of cytokines in the population. Mitochondria are considered as the prime targets for arsenic toxicity, these cytokines have been found to be involved in the generation of free radicals via mitochondrial respiratory chain reaction (Volk et al. 2000). Further, the pro-inflammatory cytokines including TNF-α, IL-1β and IL-6 are also secrets due to the arsenic toxicity and generates inflammatory responses (Duramad et al. 2007; Singh et al. 2010a, b; Das et al. 2012). These pro-inflammatory mediators are involved in the various biological and cellular comebacks including tumor progression, growth factor, transcription factor and activation of pro-apoptotic proteins (Manna et al. 2000). Studies have been showed that arsenic exposure increased the incidence of autoimmune mediated diseases, increased expression of tumor necrosis factor-α (TNFα) and Interleukin-8 (IL-8) (Bohler et al. 2000; Tseng 2004). Arsenic exposure in mammals impairs the activities of lymphocyte, monocyte, macrophage and affects the cellular immune responses resulting in immunosuppression (Burchiel et al. 2009; Raqib et al. 2009; Qian et al. 2010). In the present study, arsenic treatment in mice significantly increased the levels of TNF-α, serum Interleukin-1 beta and Interleukin-6. It also increased the relative frequency of CD8+(Tc) cells sub-population and decreased CD4+(Th), T (CD3) and B (CD19) cells as compared to controls.

Studies have been reported the protective efficacy of herbal extracts and synthetic agents in arsenic induced oxidative damage and inflammation (Flora and Gupta 2007; Sharma et al. 2009; Yadav et al. 2009; Singh et al. 2013, 2014a; Srivastava et al. 2014). Amla is widely accepted as an immune enhancer and have multiple pharmacological and immunomodulatory properties due to the presence of various phenolics and its derivatives (SaiRam et al. 2002; Sharma et al. 2009; Sreeramulu and Raghunath 2009; Singh et al. 2013). Toxicity produced through exposure of ethanol, carbon tetrachloride, or hexachlorocyclohexane have been found to be protected following treatment with amla (Pramyothin et al. 2006; Anilkumar et al. 2007). Oxidative effects of arsenic have been found to be counteracted by plant extracts and pharmacological agents due to their antioxidant potential and metal binding property (Flora and Gupta 2007; Sinha et al. 2008). Amla constitutes vitamin C which mainly acts as an antioxidant compound and has binding affinity with arsenic. The deficiencies of vitamin c may cause hyperlipidaemia and liver dysfunctions (Sharma et al. 1989, 1990). Simultaneous exposure of antioxidant like vitamins C, E and some essential metal such as zinc has been found to be beneficial in increasing arsenic mobilization and assisting the recovery of altered biochemical levels during chelating therapy (Kannan and Flora 2004; Modi et al. 2005). We have recently reported the immunomodulatory and anti-oxidative potential of amla against arsenic induced oxidative stress and apoptosis in thymocytes and spleenocytes of mice (Singh et al. 2013, 2014a). In the present study simultaneously treatment with arsenic and amla significantly inhibited the serum urea, glucose, TG and levels of TNF-α, IL-1β and Interleukin-6 in serum. At the same time the arsenic induced alterations in CD8+ and CD4+ cells and damage in B and T cells population has been found to be recovered following simultaneous treatment with arsenic and amla as compared to those treated with arsenic alone. One of the most interesting observations in our study is the ability of amla to reduce the concentration of arsenic in the thymus and spleen tissue through decreased arsenic burden in target tissues by metal chelating properties which may play a crucial role in its immunoprotective effect.

## Conclusions

We can conclude from the present study that arsenic induced immunotoxicity linked with inflammation has been significantly protected through simultaneous treatment with arsenic and amla that was due to anti-inflammatory and antioxidant activity of amla. The metal binding property of amla could reduce the load of arsenic in spleen and thymus and help to decrease the generation of reactive oxygen and nitrogen species associated with the oxidative stress and imparts its protective effects. Amla may modulate inflammatory responses by decreasing the production of several chemokines, preventing infiltration of immune cells to the damaged area, and promoting the generation of immunoprotective cytokines to accelerate the repair process. As the mechanism of prevention is not clearly understood, further studies are required to investigate the detailed mechanism of immunoprotection of amla in arsenic induced immunotoxicity.

## Additional file

**Additional file 1.** **Table S1.** The effect of arsenic, amla and their co-treatment on the assessment of Th/Tc (CD4+/CD8+) cells ratio in thymocytes and T and B cells in splenocytes of mice are presented. **Table S2.** Assessment of arsenic level in thymus and spleen of mice after the treatment of arsenic, amla and co-treatment are presented.
