# Supplementary material for: Protective effect of Emblica-officinalis in arsenic induced biochemical alteration and inflammation in mice
Source: Springerplus. 2015 Aug 21;4:438. doi: 10.1186/s40064-015-1227-9 (PMC4545902; doi:10.1186/s40064-015-1227-9)
Supplement: Supplementary file 1 — Additional file 1. Table S1. The effect of arsenic, amla and their co-treatment on the assessment of Th/Tc (CD4+/CD8+) cells ratio in thymocytes and T and B cells in splenocytes of mice are presented. Table S2. Assessment of arsenic level in thymus and spleen of mice after the treatment of arsenic, amla and co-treatment are presented. [file 40064_2015_1227_MOESM1_ESM.pptx]

## Slide 1
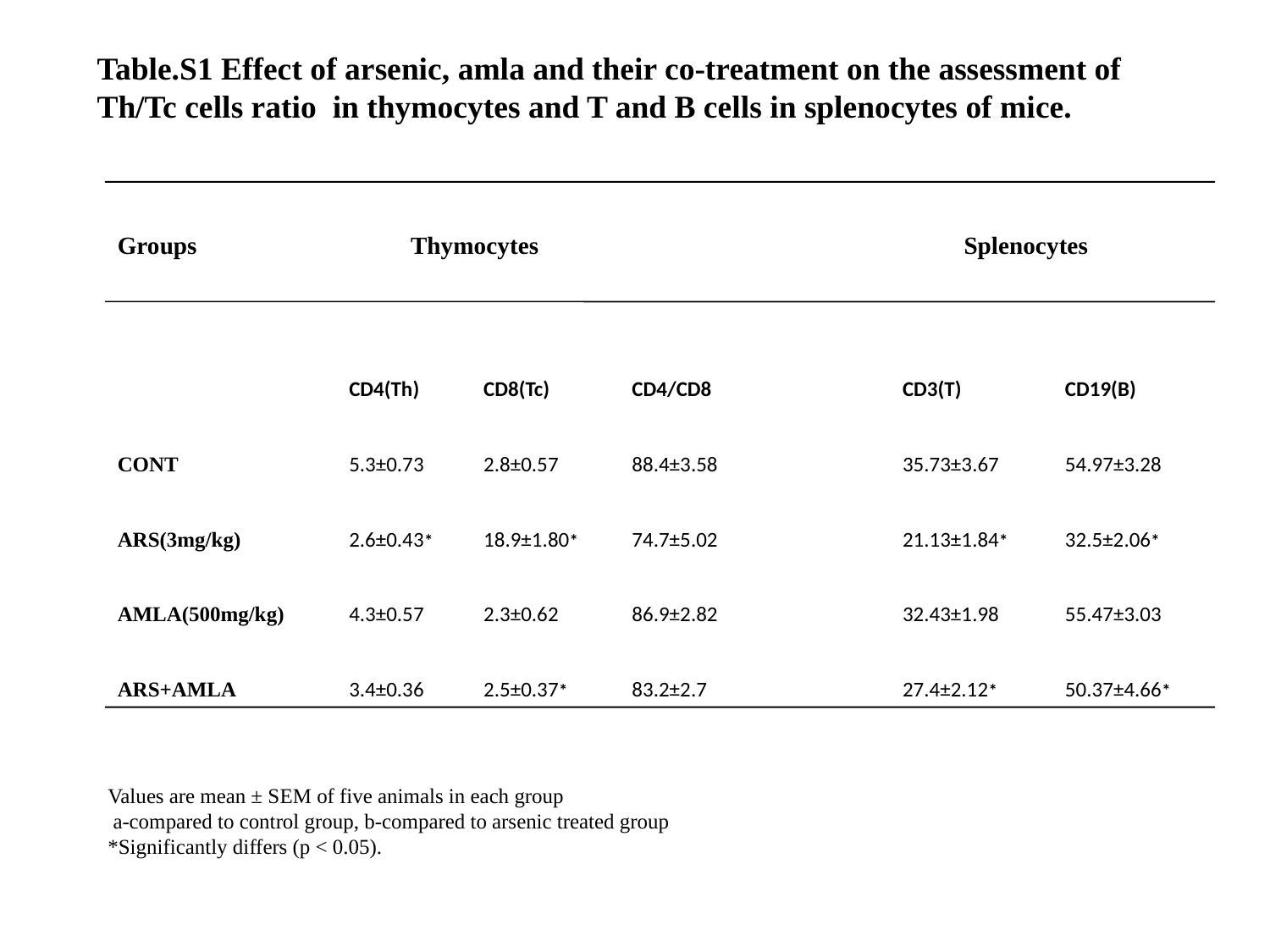

Table.S1 Effect of arsenic, amla and their co-treatment on the assessment of Th/Tc cells ratio in thymocytes and T and B cells in splenocytes of mice.
Groups
 Thymocytes
 Splenocytes
CD4(Th)
CD8(Tc)
CD4/CD8
CD3(T)
CD19(B)
CONT
5.3±0.73
2.8±0.57
88.4±3.58
35.73±3.67
54.97±3.28
ARS(3mg/kg)
2.6±0.43*
18.9±1.80*
74.7±5.02
21.13±1.84*
32.5±2.06*
AMLA(500mg/kg)
4.3±0.57
2.3±0.62
86.9±2.82
32.43±1.98
55.47±3.03
ARS+AMLA
3.4±0.36
2.5±0.37*
83.2±2.7
27.4±2.12*
50.37±4.66*
Values are mean ± SEM of five animals in each group
 a-compared to control group, b-compared to arsenic treated group
*Significantly differs (p < 0.05).

## Slide 2
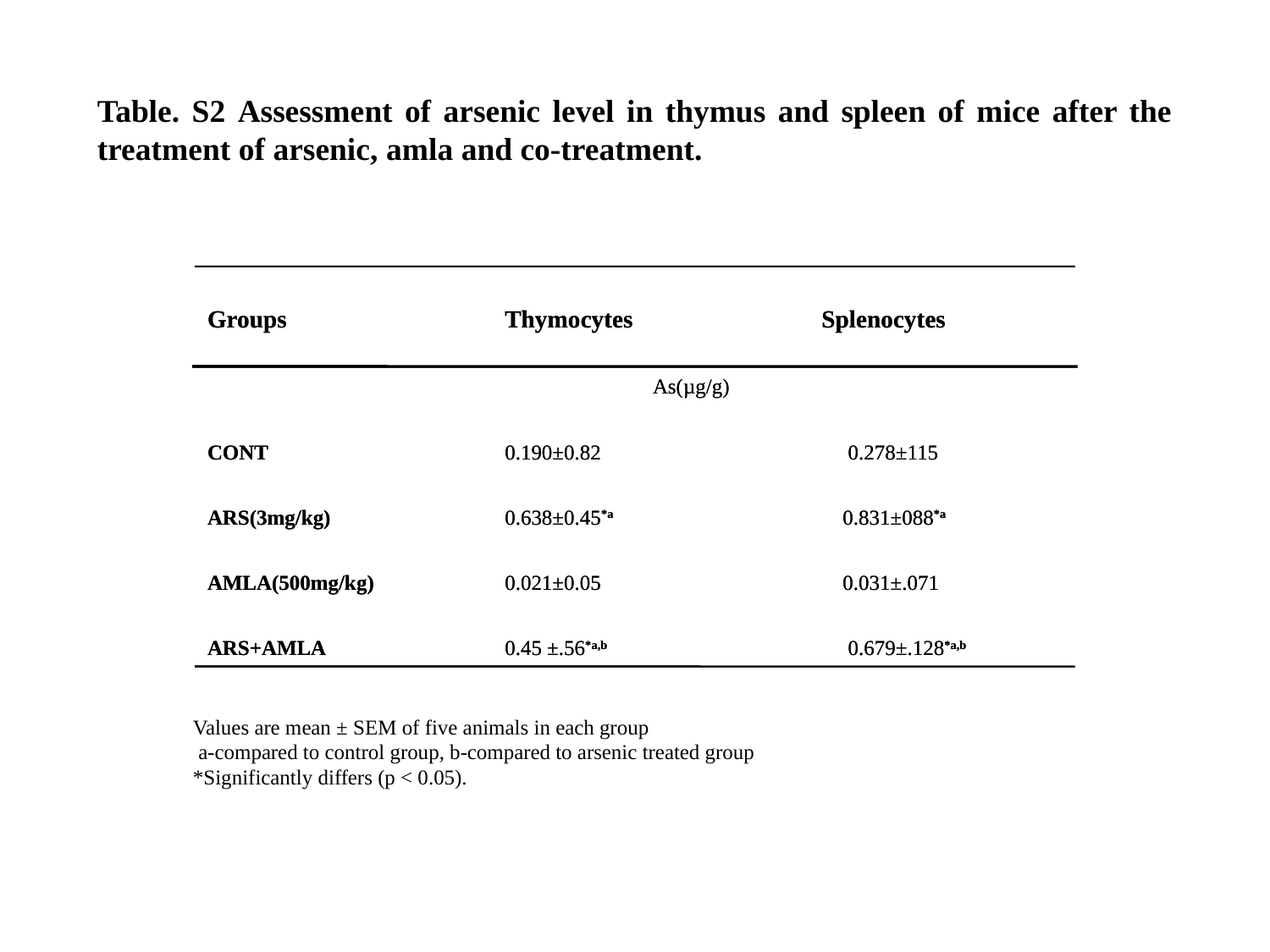

Table. S2 Assessment of arsenic level in thymus and spleen of mice after the treatment of arsenic, amla and co-treatment.
Groups
Groups
Thymocytes
Thymocytes
Splenocytes
Splenocytes
 As(µg/g)
 As(µg/g)
CONT
CONT
0.190±0.82
0.190±0.82
 0.278±115
 0.278±115
ARS(3mg/kg)
ARS(3mg/kg)
0.638±0.45*a
0.638±0.45*a
 0.831±088*a
 0.831±088*a
AMLA(500mg/kg)
AMLA(500mg/kg)
0.021±0.05
0.021±0.05
 0.031±.071
 0.031±.071
ARS+AMLA
ARS+AMLA
0.45 ±.56*a,b
0.45 ±.56*a,b
 0.679±.128*a,b
 0.679±.128*a,b
Values are mean ± SEM of five animals in each group
 a-compared to control group, b-compared to arsenic treated group
*Significantly differs (p < 0.05).
